# Liquor Drinking

**Published:** 1856-11

**Authors:** 


### LIQUOR DRINKING,

As an habitual thing, not only impairs the health of the drinkers themselves, but entails scrofulous disease on their children as to body, and imbecility as to mind; as witness of the latter, Asylum reports are full abundant; and of the former, every day's observation tells the tale.

It is useful, therefore, to inquire, and to point out, now and then, some of the ways in which drunkards are made. An impressive incident is given in the *New York Evening Post*, which ought not to be permitted to perish with a daily paper.

A gentleman, a few months married, on coming home one evening, tired and depressed from a long summer day's toil, having dined in his office from press of business, found his young wife in a rocking-chair, slip-shod, in a soiled morning-gown, one leg over the knee, reading a novel.

"Why Fanny, not dressed yet! what have you been doing all day?"

"O, I have been reading this book, and it is so interesting; there is only one chapter more. Please ring the tea-bell; I am so tired, and it is too warm to be dressed up."

"But before we were married, I never found you not dressed."

"O! then I dressed according to the company, and do so still."

Being discomposed, he thought he would take a short walk to dissipate his unpleasant feelings, and soon passing a cheery, well-lighted room, he entered. It was a debating-club; he found several of his acquaintances there, *all married men!* Falling into conversation with them, the evening passed rapidly.

It was a week before he spent another evening out. But being annoyed at the continued slovenliness of his wife, he left her to her novel and slip-shod shoes, and became a regular attendant at the debating-room, the "exercises" of which, uniformly closed with various mixtures of brandy and water for purposes of imbibition. In due time, the once exemplary husband became a hard drinker.

We know a case of some resemblance:

An up-town gentleman, living in his own house, who never went out alone after tea, had been greatly pressed all day in meeting some bank calls, which to him were heavy and unusual. He came home late in the evening in a state of exhaustion. And most unusual for him, he did not go down to tea, but stretching himself on the sofa, and feeling as if he were about to have a chill, asked his wife, who was sitting by the fire, if there was any such thing as brandy in the house; and if so, he would like to have a glass of brandy and water. She left her seat, saying she was very tired, but would get some for him. After waiting a full half hour by the clock, she returned, saying she had been talking with the cook about to-morrow's dinner, but that she would get the toddy if it was still wanted. Feeling anxious to keep off the chill, and not wishing further delay, he said to her it was of no consequence; and taking his hat, went into the street, and stepping into the first grocery he came to, for the first time in his life, paid for a glass of liquor. It was just dark as he came out of the den, but the chill came on him in the street, with several days' sickness succeeding.

Whether that man will die in the gutter, a sot and an outcast, no mortal can tell. But if he does, it is not difficult to answer the pregnant inquiry of the *Evening Post*,

### Who's to Blame?

While we do not deny that men fall into bad practices from want of principle and from yielding themselves to the gratification of evil appetites and passions, it cannot be denied, that pecuniary, moral, social and domestic ruin, is properly laid at the door of a wife, who, as a girl, had two curses:

First, The curse of an education at a fashionable boarding school or "Institute."

Second, The curse of having means to revel in novel reading.

And we wish here to express our fullest conviction, that Female Boarding Schools, *as generally conducted,* are properly denounced by some of the best medical writers, as the hot beds of moral corruption and physical degeneration; and that they wholly unfit their pupil, for the positions which they are destined to occupy in subsequent life.

